# Activity of the genus *Zanthoxylum* against diseases caused by protozoa: A systematic review

**DOI:** 10.3389/fphar.2022.873208

**Published:** 2023-01-09

**Authors:** Juliana Correa-Barbosa, Daniele Ferreira Sodré, Pedro Henrique Costa Nascimento, Maria Fâni Dolabela

**Affiliations:** ^1^ Pharmaceutical Science Post-graduation Programx, Federal University of Pará, Belém, Pará, Brazil; ^2^ Faculty of Pharmacy, Federal University of Pará, Belém, Brazil

**Keywords:** Malaria, Leishmaniasis, Chagas disease, sleeping sickness, *Zanthoxylum*

## Abstract

Neglected diseases (NDs) are treated with a less varied range of drugs, with high cost and toxicity, which makes the search for therapeutic alternatives important. In this context, plants, such as those from the genus *Zanthoxylum*, can be promising due to active substances in their composition. This study evaluates the potential of species from this genus to treat NDs. Initially, a protocol was developed to carry out a systematic review approved by Prospero (CRD42020200438). The databases PubMed, BVS, Scopus, Science Direct, and Web of Science were used with the following keywords: “zanthoxylum,” “xanthoxylums,” “fagaras,” “leishmaniasis,” “chagas disease,” “malaria,” and “African trypanosomiasis.” Two independent evaluators analyzed the title and abstract of 166 articles, and 122 were excluded due to duplicity or for not meeting the inclusion criteria. From the 44 selected articles, results of *in vitro*/*in vivo* tests were extracted. *In vitro* studies showed that *Z. rhoifolium*, through the alkaloid nitidine, was active against *Plasmodium* (IC50 <1 μg/ml) and *Leishmania* (IC50 <8 μg/ml), and selective for both (>10 and >30, respectively). For Chagas disease, the promising species (IC50 <2 μg/ml) were *Z. naranjillo* and *Z. minutiflorum*, and for sleeping sickness, the species *Z. zanthoxyloides* (IC50 <4 μg/ml) stood out. In the *in vivo* analysis, the most promising species were *Z. rhoifolium* and *Z. chiloperone*. In summary, the species *Z. rhoifolium*, *Z. naranjillo*, *Z. minutiflorum*, *Z. zanthoxyloides*, and *Z. chiloperone* are promising sources of active molecules for the treatment of NDs.

## 1 Introduction

Neglected diseases (NDs), also called neglected tropical diseases (NTDs), are highly prevalent infectious conditions marked by a high degree of morbidity, mainly in the poorest and most vulnerable populations. These occur in developing countries, mostly in Africa, Asia, and the Americas (Souza, 2010; Sousadas et al., 2019).

Several NDs are caused by protozoa: malaria is caused by *Plasmodium*; leishmaniasis, by *Leishmania*; Chagas disease, by *Trypanosoma cruzi*, and sleeping sickness is caused by *Trypanosoma brucei*. *Plasmodium falciparum*, *P. vivax*, *P. ovale*, *P. malariae*, and *P. knowlesi* infect vertebrate erythrocytes and can cause severe and non-severe malaria (Bannister and Sherman, 2009). In the genus *Leishmania*, there are 30 species that infect mammals, 21 of them affect humans, and transmission occurs through the bite of female sandflies (Bates, 2007).

The species *Trypanosoma cruzi* and its different strains are responsible for causing Chagas disease, which is transmitted by vectors (80% of cases), blood transfusion (5–20% of cases; Fidalgo et al., 2018), and orally (Ferreira et al., 2014). *Trypanosoma brucei* is the causative agent of the African sleeping sickness, which occurs in 36 countries in sub-Saharan Africa, the poorest region in the world (Matthews, 2005).

For the treatment of Chagas disease, only two drugs are available, and they can cause severe adverse events (BRASIL, 2018). Also, in leishmaniasis, the number of drugs available for its treatment is limited (Comandolli-Wyrepkowski et al., 2020), while for malaria there are limitations of drugs to treat the hepatic form and resistant strains of *P. falciparum* (ANVISA, 2020).

Moreover, the treatment of diseases caused by protozoa has a high therapeutic cost, low adherence to treatment, and high inefficiency, since protozoa have developed resistance to the available drugs (Belloze, 2013), damaging to public health. In this sense, it is urgently necessary to search for new therapeutic alternatives, drugs that can cure such illnesses at a low cost with high levels of effectiveness. Thus, new drugs are needed for diseases caused by protozoa, and some studies have already highlighted the use of plants as a source of antiprotozoal agents (Ohashi et al., 2018).

Different studies have evaluated the biological activity of medicinal species or their toxicity, generating isolated information. The systematic review allows the integration of these results and demonstrates their therapeutic potential (Goodman et al., 2016; Melo-Neto et al., 2016). A genus that already has some chemical, pharmacological, and toxicity studies is *Zanthoxylum* (Dofuor et al., 2019b; Da Silva et al., 2019). From this genus (*Zanthoxylum*), there are reports of pharmacological evaluation of extracts (Adia et al., 2016), fractions (Alam and Najam us Saqib, 2017), and isolated substances (Bouquet et al., 2012) confirmed by *in vitro/in vivo* studies. The alkaloid class is the most commonly described, with some studies proving its antiprotozoal activity.

The hypothesized mechanism of action of the alkaloid class is as follows: cytoskeletal blockage or depolymerization (Fernandes, 2017), direct binding to the heme group of hemoglobin, inhibition of vacuolar phospholipase, protein synthesis inhibition, and interaction with DNA (França et al., 2008). Therefore, this study was carried out to compile and evaluate the *in vitro/in vivo* activities of the genus *Zanthoxylum* against diseases caused by protozoa.

## 2 Methods

### 2.1 Selection criteria and search strategies

**FIGURE 1:**
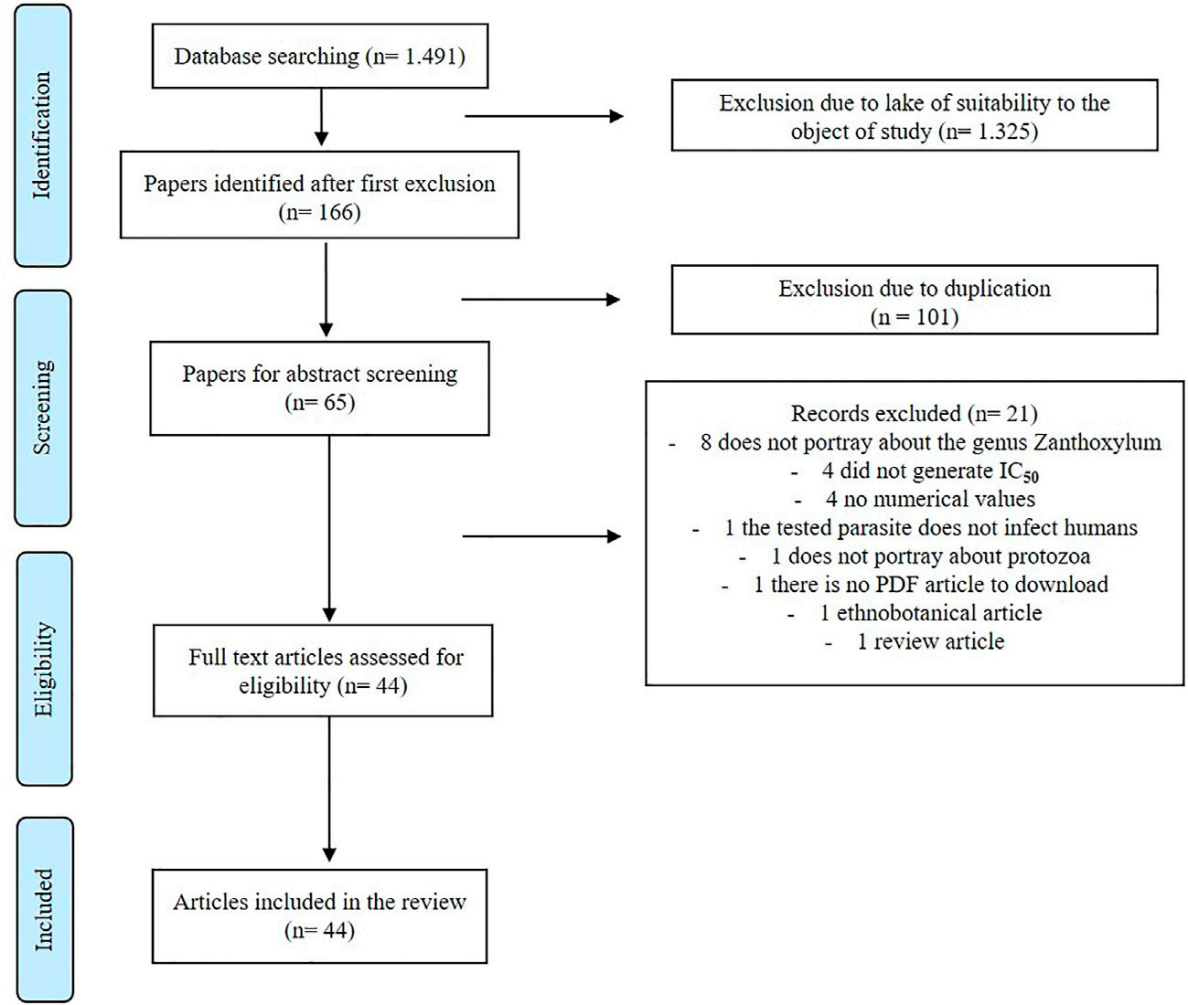
Article selection process.

For the development of this study, all original indexed articles, written in English, Portuguese, and Spanish, which reported data about the activities of extracts, fractions, and isolated compounds from the genus *Zanthoxylum* against *Leishmania*, *Plasmodium*, *Trypanosoma cruzi*, and *Trypanosoma brucei* on preclinical experiments (*in vitro* and/or *in vivo*) were included. Review articles, book chapters, case studies, and articles about genus activity against protozoan vectors and activities of synthesized compounds were excluded (Figure 1). Initially, a protocol was developed to carry out the systematic review, and this was approved by Prospero (CRD42020200438).

The electronic databases PubMed, Virtual Health Library (VHL), Scopus, Science Direct, and Web of Science, all free access databases, were used to search for articles. The search descriptors were combinations of terms found in the titles of medical subjects (Mesh) and Descriptors in Health Sciences (DeCs). The date of the last search was May 2020.

In PubMed (pubmed.ncbi.nlm.nih.gov), there was mainly North American literature, and the descriptors used were as follows: (zanthoxylum) OR (zanthoxylums)) OR (xanthoxylum)) OR (xanthoxylums)) OR (fagaras, Zanthoxylum)) AND ((((Leishmaniasis) OR (Chagas disease)) OR (malaria)) OR (African trypanosomiasis)). In the BVS (bvsalud.org), there was a predominance of literature from Latin America when the following terms were used: (((leishmaniose) OR (doença de chagas) OR (malaria) OR (tripanossomíase Africana))) AND (tw: ((zanthoxylum)) OR (xanthoxylum).

In the Scopus database (scopus.com), which includes several knowledge areas, we used the following descriptors: (zanthoxylum OR xanthoxylum OR “fagaras, Zanthoxylum”) AND ALL (leishmaniasis OR “Leishmania Infection” OR “Chagas disease”" OR “Trypanosoma cruzi Infection” OR malaria OR “Plasmodium Infection” OR “African trypanosomiasis” OR “African Sleeping Sickness").

Operated by the Anglo-Dutch publisher Elsevier, Science Direct (sciencedirect.com) was also included in the research and the descriptors were as follows: (zanthoxylum OR xanthoxylum) AND (leishmaniasis OR Leishmania Infection OR Chagas disease OR Trypanosoma cruzi Infection OR malaria OR Plasmodium infection OR African trypanosomiasis). Finally, we included Web of Science (webofscience.com) as a multidisciplinary base and the research adopted the descriptors (zanthoxylum OR xanthoxylum OR fagaras, Zanthoxylum”) AND (leishmaniasis OR chagas disease” OR malaria OR african trypanosomiasis").

The articles were selected by two independent examiners, based on the reading of the title and abstract, with a third final examiner. Potentially eligible articles were read in full.

### 2.2 Bias risk

Because the selected studies may present risks of bias, two instruments were used: the guidelines for pre-clinical *in vitro* reports on dental materials, developed by Faggion et al. (2012) modified to meet the criteria of *in vitro* studies (Table 1), and SYRCLE, developed by Hooijmans et al. (2014) to assess the risk of bias of *in vivo* studies (Table 2). The studies were analyzed in pairs by two examiners, with a third evaluator when there was disagreement.

**TABLE 1:** Results of *in vitro* study evaluations.

Introduction		Methodology								Results and discussion					
I1	I2	I3	I4	I5	I6	I7	I8	I9	I10	I11	I12	I13	I14	Total	Author (year)
1	1	1	1	1	0	0	0	0	1	1	1	1	0	8	Adia et al. (2016)
0.5	1	1	1	1	0	0	0	0	1	1	1	0	0	7.5	Alam and Najam us Saqib (2017)
1	1	1	1	1	0	0	0	0	1	1	1	1	0	9	Aratikatla et al. (2017)
1	1	1	1	1	0	0	0	0	1	1	1	1	0	9	Bertani et al. (2005)
1	1	1	1	1	0	0	0	0	1	1	1	1	0	9	Bouquet et al. (2012)
1	0.5	0.5	1	0.5	0	0	0	0	1	1	0.5	0	0	6	Castillo et al. (2014)
1	1	1	1	1	0	0	0	0	1	1	1	1	0	9	Cerbrián-Torrejón et al. (2011)
1	1	1	1	1	0	0	0	0	1	1	1	1	0	9	Chavez et al. (2014)
0.5	1	0.5	1	1	0	0	0	0	1	0.5	0.5	0.5	0	5.5	Chinchilla-Carmona et al. (2012)
0.5	1	1	1	1	0	0	0	0	1	1	0.5	1	0	8	Costa et al. (2018)
1	1	1	1	1	0	0	0	0	1	1	0.5	1	0	8.5	Da Silva et al. (2019)
1	1	1	1	1	0	0	0	0	1	1	1	0	0	8	Dofuor et al. (2019a)
1	1	1	1	1	0	0	0	0	1	1	1	0	0	8	Dofuor et al. (2019b)
1	1	1	0.5	1	0	0	0	0	0.5	0.5	1	0	0	6.5	Freiburghaus et al. (1996)
1	1	1	1	1	0	0	0	0	1	1	1	1	0	9	Gansane et al. (2010)
1	1	1	1	1	0	0	0	0	1	1	1	1	0	9	Gansane et al. (2010)
1	1	1	1	1	0	0	0	0	1	1	1	1	0	9	Gessler et al. (1994)
1	1	0.5	1	1	0	0	0	0	1	1	0.5	1	0	8	Goodman et al. (2016)
1	1	0.5	1	1	0	0	0	0	1	0.5	1	1	0	8	Goodman et al. (2019)
1	1	1	1	1	0	0	0	0	1	1	1	0	0	8	Jullian et al. (2006)
1	1	1	1	1	0	0	0	0	1	1	1	1	0	9	Kirira et al. (2006)
1	1	1	1	1	0	0	0	0	1	1	1	1	0	9	Kamanzi Atindehou et al. (2004)
1	1	1	1	1	0	0	0	0	1	1	1	0	0	8	Ledoux et al. (2018)
1	0.5	1	1	1	0	0	0	0	1	1	0.5	1	0	8	Lima (2015)
1	0.5	1	1	1	0	0	0	0	1	1	0.5	1	0	8	Mafezoli et al. (2000)
1	1	1	1	1	0	0	0	0	1	1	1	0	0	8	Muganga et al. (2010)
1	0.5	1	1	1	0	0	0	0	1	1	0.5	1	0	8	Muganga et al. (2014)
1	0.5	1	1	1	0	0	0	0	1	1	0.5	1	0	8	Muthaura et al. (2015)
1	1	1	1	1	0	0	0	0	1	1	1	0	0	8	Nibret et al. (2010)
1	1	1	1	1	0	0	0	0	1	1	1	1	0	9	Ohashi et al. (2018)
1	1	1	1	1	0	0	0	0	1	1	1	1	0	9	Penali et al. (2007)
1	1	0.5	1	1	0	0	0	0	1	1	1	0	0	7.5	Randrianarivelojosia et al. (2003)
1	1	1	1	1	0	0	0	0	1	1	1	1	0	9	Rukunga et al. (2009)
1	1	1	1	1	0	0	0	0	1	1	1	1	0	9	Sandjo et al. (2016)
1	1	0.5	1	1	0	0	0	0	1	1	0.5	0	0	7	Stangelang et al. (2010)
0.5	1	1	1	1	0	0	0	0	1	1	1	1	0	8.5	Tanoh et al. (2020)
1	1	1	1	1	0	0	0	0	1	1	1	1	0	9	Tchinda et al. (2009)

I1: abstract; I2: introduction and objectives; I3: methods; I4: results; I5: sample size; I6: randomization; I7: allocation concealment; I8: implementation; I9: blindness; I10: statistical methods; I11: results and estimates; I12: discussion; I13: other information; and I14: protocol.

**TABLE 2:** Results of *in vivo* studies.

Selection bias			Performance bias		Detection bias		Friction bias	Reporting bias	Other sources of bias	
Sequence generation	Characteristic base	Allocation concealment	Random housing	Blinding	Results evaluation	Blinding	Incomplete result data	Results report	Others	Author (year)
I1	I2	I3	I4	I5	I6	I7	I8	I9	I10	
N	N	N	N	N	N	N	M	M	N	Enechi et al. (2019)
N	N	N	N	N	N	N	N	N	N	Ferreira et al. (2007)
N	N	N	N	N	N	N	N	M	N	Ferreira et al. (2011)
N	N	N	N	N	N	N	N	S	N	Muganga et al. (2014)
N	N	N	N	N	N	N	N	S	N	Musila et al. (2013)
N	N	N	N	N	N	N	N	N	N	Bertani et al. (2005)
N	N	N	N	N	N	N	N	S	N	Ferreira et al. (2002)
N	N	N	N	N	N	N	N	M	N	Castillo et al. (2014)
N	N	N	N	N	N	N	N	N	N	Bouquet et al. (2012)
N	N	N	N	N	N	N	N	N	N	Were et al. (2010)

N: unsatisfactory criterion; S: satisfied criterion; M: irresolute or indeterminate criterion.

Based on the material developed by Faggion et al. (2012), the risk of bias was classified into numbers, with the value one representing a low risk of bias; 0.5, potential risk of bias; and 0, risk of bias, as shown in Table 1. This instrument was chosen because it is an easy-to-handle tool with a high intra-examiner agreement. The analysis was based on the following aspects: 1) abstract; 2) introduction, background, and objectives; 3) methods and intervention; 4) results; 5) sample size; 6) randomization: sequence generation; 7) allocation concealment; 8) implementation; 9) blinding (10) statistical methods; 11) results and estimates 12) discussion; 13) other information (financing); and 14) protocol.

The tool developed by Hooijmans et al. (2014) was created specifically for intervention studies in animals and is based on the Cochrane tool. The risk of bias was classified into specific characters, where N (not relevant to the item); S (showed relevance to the item); and M (presented medium relevance to the item). The analysis was based on the aspects (1; 2; 3) selection bias; (4; 5) performance bias (6; 7) detection bias; 8) friction bias; 9) random bias; and (10) other sources of bias (Table 2).

Regarding the activity classification, the parameters of Dolabela et al. (2015) for the genus *Plasmodium* and those of Mota et al. (2015) for the genus *Leishmania* were used, and the following parameters were adopted to evaluate the activity against *Trypanosoma* *cruzi* and *Trypanosoma brucei*: IC_50_ < 10 μg/ml, good activity; IC_50_ of 10–50 μg/ml, moderate activity; IC_50_ 50–100 μg/ml, low activity; and IC_50_ > 100 μg/ml, inactive. For the selectivity index (IS), the one calculated from the ratio between cytotoxicity for macrophages (CC_50_) and activity against amastigotes (IC_50_) was used. IS values >20.0 indicate that the sample tested was more toxic to the parasite than to the host cell. IS values <20.0 demonstrate toxicity of the compound, adapted as described by Don and Ioset (2014).

### 2.3 Data extraction

Variables of interest for *in vitro* studies (first author, year of study, genus, species, part of the plant used, 50% inhibitory concentration—IC_50_, evolutionary form, protozoan species, 50% cytotoxic concentration—CC_50_, cell lineage, selectivity index, and activity classification) were transferred to tables in Word (Tables 3, 5, 7, 9).

Variables of interest for *in vivo* studies (first author, year of study, genus, species, part of the plant used, % parasitemia, % chemosuppression, % inhibition of the protozoan, protozoan species, animal species, 50% cytotoxic concentration (CC_50_), cell lineage, and selectivity index) were transferred to tables in Word (Tables 4, 6, 8).

## 3 Results

### 3.1 Selection of articles

In the search, 1,491 articles were identified, of which only 166 met the inclusion criteria. In the screening phase, the authors evaluated the titles of these 166 articles. Of these, 101 articles were excluded due to duplicity, and therefore, 65 articles were chosen for reading. After analyzing the abstracts, eight articles were excluded because they did not study the genus *Zanthoxylum*, four because they did not generate the IC_50_, four because they did not present numerical values, one because the tested parasite did not infect humans, one because it did not portray protozoa, one because it was not in a document available for reading without open access, one for being ethnobotanical article, and one review article, totaling 21 articles excluded. Thus, 44 original articles were included in this systematic review (Figure 1).

In the introduction and initial part of the methodology, all articles met the verification completely or partially, noting that the first items are in accordance with the methodology adapted from Faggion Jr et al. (2012). However, the verification in the final part of the methodology is totally different from the initial one, as none of the articles meet the guideline verification criteria, and this is a warning as to how the methodologies of these *in vitro* articles are being managed (Table 1).

However, based on the results and discussion, it is possible to notice again the improvement in the verification of these articles, and they fully or partially meet this verification. However, in the last item (protocol), none of the articles meet the verification criteria. This means that the articles did not expose their protocols, which hinders the reproducibility of this work (Table 1).

In the end, to have a quality overview of these articles, a score was generated according to the items the article presented during the methodology verification, and after that, we verified that the studies by Aratikatla et al. (2017), Bertani et al. (2005), Bouquet et al. (2012), Cebrián-Torrejón et al. (2011), Enciso et al. (2014), Gansane et al. (2010), Gessler et al. (1994), Kirira et al. (2006), Kamanzi Atindehou et al. (2004), Ohashi et al. (2018), Penali et al. (2007), Rukunga et al. (2009), Sandjo et al. (2016), and Tchinda et al. (2009) and obtained the highest score (score 9 out of 14) among the 37 articles evaluated by this methodology (Table 1).

None of the articles evaluated by Hooijmans et al. (2014) met the satisfaction criteria in selection, performance, and detection biases. Only the studies by Bertani et al. (2005), Musila et al. (2013), and Muganga et al. (2014) showed satisfaction in the assessment used in the reporting bias (Table 2).

### 3.3 Data collection

An extensive literature review was carried out to identify whether extracts, fractions, and pure substances from the genus *Zanthoxylum* presented *in vitro* and *in vivo* activity against protozoa of the genus *Plasmodium*, *Leishmania*, and *Trypanosoma*. The results can be seen in Tables 3, 4, 5, 6, 7, 8, 9.

**TABLE 3:** *In vitro* antiparasitic activity against *Plasmodium.*

Species	Extract, fraction, and isolated substance	Przotozoan activity (IC_50_ µg/mL ± SD)		Cytotoxicity (CC_50_ µg/mL ± SD)	Cell line/crustaceans	Selectivity index (SI)	A. C. (Dolabela et al. 2015)	Author (year)
		Resistant	Sensitive					
*Plasmodium falciparum*								
*Z. rhoifolium*	Ext. H_2_O (b)	W2: >11	ND	ND	ND	ND	MA/I	Bertani et al. (2005)
*Z. chiloperon*	Ext. CH_2_CL_2_ (sb)	K1: 8.9	F32: 8.9	12.3	MRC5	1.3	A	Cerbrián-Torrejón et al. (2011)
*Z. chiloperon*	Ext. EtOH (sb)	K1: 9.3	F32: 10.5	13.0	MRC5	1.3	A	Cerbrián-Torrejón et al. (2011)
*Z. chiloperon*	Ext. MeOH (sb)	K1: >100	F32: 89.5	>100	MRC5	>1.1	K1: I/F32: MA	Cerbrián-Torrejón et al. (2011)
*Z. zanthoxyloides*	Ext. MeOH (sb)	W2: 40.9	ND	31.1	K562S	0.8	MA	Gansane et al. (2010)
*Z. zanthoxyloides*	Ext. MeOH/H_2_O (sb)	W2: 13.6	ND	>125	K562S	>9.2	MA	Gansane et al. (2010)
*Z. zanthoxyloides*	Ext. CH_2_CL_2_ (sb)	W2: 16.2	ND	4.7	K562S	0.3	MA	Gansane et al. (2010)
*Z. zanthoxyloides*	Ext. H_2_O (sb)	W2: >50	ND	>125	K562S	>2.5	MA/I	Gansane et al. (2010)
*Z. zanthoxyloides*	Ext. CH_2_CL_2_ (sb)	ND	3D7: 112.15 ± 0.01	583.53 ± 0.02	Jurkat	5.20	I	Dofuor et al. (2019a)
*Z. zanthoxyloides*	Ext. EtOH (sb)	ND	3D7: 112.15	ND	ND	ND	I	Ohashi et al. (2018)
*Z. zanthoxyloides*	Ext. EtOH (rb)	ND	3D7: 334.77	ND	ND	ND	I	Ohashi et al. (2018)
*Z. zanthoxyloides*	Ext. EtOH (l)	ND	3D7: >1,000	ND	ND	ND	I	Ohashi et al. (2018)
*Z. zanthoxyloides*	FA 1 (sb)	W2: 1.2	ND	24.1	K562S	20.7	A	Gansane et al. (2010)
*Z. zanthoxyloides*	FA 2 (sb)	W2: 9.10	ND	30.72	K562S	3.38	A	Gansane et al. (2010)
*Z. zanthoxyloides*	FA 3 (sb)	W2: 2.44	ND	12.44	K562S	5.10	A	Gansane et al. (2010)
*Z. zanthoxyloides*	FA 4 (sb)	W2: 1.91	ND	11.74	K562S	6.15	A	Gansane et al. (2010)
*Z. zanthoxyloides*	FA 5 (sb)	W2: 4.32	ND	13.11	K562S	3.03	A	Gansane et al. (2010)
*Z. zanthoxyloides*	FA 6 (sb)	W2: 21.36	ND	18.83	K562S	0.90	MA	Gansane et al. (2010)
*Z. zanthoxyloides*	FA 7 (sb)	W2: 24.88	ND	15.7	K562S	0.63	MA	Gansane et al. (2010)
*Z. zanthoxyloides*	FA 8 (sb)	W2: 10.14	ND	21.11	K562S	2.08	MA	Gansane et al. (2010)
*Z. zanthoxyloides*	FA 9 (sb)	W2: 11.26	ND	9.94	K562S	0.90	MA	Gansane et al. (2010)
*Z. zanthoxyloides*	FA 10 (sb)	W2: 5.00	ND	5.44	K562S	1.09	A	Gansane et al. (2010)
*Z. zanthoxyloides*	FA 11 (sb)	W2: 24.10	ND	22.28	K562S	0.93	MA	Gansane et al. (2010)
*Z. zanthoxyloides*	FA 12 (sb)	W2: 8.62	ND	13.75	K562S	1.60	A	Gansane et al. (2010)
*Z. chalybeum*	Ext. EtOAc (sb)	FCR3: 0.57 ± 0.39	NF54: 3.21 ± 0.23	ND	ND	ND	VA	Adia et al. (2016)
*Z. chalybeum*	Ext. EtOH (sb)	K1: 31	ND	ND	ND	ND	MA	Gessler et al. (1994)
*Z. chalybeum*	Ext. PE (sb)	K1:42	ND	ND	ND	ND	MA	Gessler et al. (1994)
*Z. chalybeum*	Ext. EtOAc (sb)	K1: 13	ND	ND	ND	ND	MA	Gessler et al. (1994)
*Z. chalybeum*	Ext. H_2_O (sb)	K1: 23	ND	ND	ND	ND	MA	Gessler et al. (1994)
*Z. chalybeum*	Ext. EtOH (rb)	K1: 2.2	ND	ND	ND	ND	A	Gessler et al. (1994)
*Z. chalybeum*	Ext. PE (rb)	K1: 10	ND	ND	ND	ND	MA	Gessler et al. (1994)
*Z. chalybeum*	Ext. EtOAc (rb)	K1: 4.2	ND	ND	ND	ND	A	Gessler et al. (1994)
*Z. chalybeum*	Ext. H_2_O (rb)	K1: 1.2	ND	ND	ND	ND	A	Gessler et al. (1994)
*Z. chalybeum*	Ext. MeOH (sb)	ND	3D7: 42.5 ± 0.4	ND	ND	ND	MA	Muganga et al. (2010)
*Z. chalybeum*	Ext. CH_2_CL_2_ (sb)	ND	3D7: 41.5 ± 0.9	ND	ND	ND	MA	Muganga et al. (2010)
*Z. chalybeum*	Ext. MeOH (rb)	W2: 1.9	3D7: 4.2 ± 2.7	40.0 ± 8.5	WI-38	9.5	A	Muganga et al. (2010)
*Z. chalybeum*	Ext. CH_2_CL_2_ (rb)	ND	3D7: 6.2 ± 0.6	ND	ND	ND	A	Muganga et al. (2010)
*Z. chalybeum*	Ext. H_2_O (rb)	ND	3D7: >50	ND	ND	ND	MA	Muganga et al. (2010)
*Z. chalybeum*	Ext. MeOH (rb)	W2: 2.9	D6: 3.7	ND	ND	ND	A	Muthaura et al. (2015)
*Z. chalybeum*	Ext. MeOH (rb)	ND	3D7: 6.18 ± 1.23	ND	ND	ND	A	Muganga et al. (2014)
*Z. chalybeum*	Ext. H_2_O (rb)	W2: 3.1	ND	ND	ND	ND	A	Muthaura et al. (2015)
*Z. chalybeum*	Ext. MeOH (rb)	ENT 30: 3.14 ± 0.28	NF54: 5.30 ± 2.82	ND	ND	ND	A	Rukunga et al. (2009)
*Z. chalybeum*	Ext. H_2_O (rb)	ENT 30: 2.32 ± 0.34	NF54: 5.52 ± 1.36	ND	ND	ND	A	Rukunga et al. (2009)
*Z. chalybeum*	Ext. H_2_O (rb)	ENT 30: 2.88 ± 0.36	NF54: 3.65 ± 0.62	ND	ND	ND	A	Rukunga et al. (2009)
*Z. chalybeum*	Ext. CH_2_CL_2_ (b)	ND	MRA-285: 2.85	ND	ND	ND	A	Stangelang et al. (2010)
*Z. chalybeum*	Ext. MeOH (b)	ND	MRA-285: 10.92	ND	ND	ND	MA	Stangelang et al. (2010)
*Z. chalybeum*	Ext. MCW (b)	ND	MRA-285: 2.72	ND	ND	ND	A	Stangelang et al. (2010)
*Z. chalybeum*	Ext. H_2_O (b)	ND	MRA-285: 3.63	ND	ND	ND	A	Stangelang et al. (2010)
*Z. chalybeum*	Ext. Act (b)	ND	MRA-285: 3.05	ND	ND	ND	A	Stangelang et al. (2010)
*Z. chalybeum*	F. CH_2_CL_2_ (rb)	ND	3D7: 4.81 ± 0.26	ND	ND	ND	A	Mugunga et al. (2014)
*Z. chalybeum*	F. H_2_O (rb)	ND	3D7: 14.37 ± 5.49	ND	ND	ND	MA	Mugunga et al. (2014)
*Z. heitzii*	Ext. Hex (b)	ND	3D7: 0.050 ± 0.004	ND	ND	ND	VA	Goodman et al. (2016)
*Z. usambarensis*	Ext. H_2_O (sb)	ENT 30: 14.33 ± 4.42	NF54: 5.25 ± 0.27	260.90 ± 1.1	A.s	18.20	MA	Kirira et al. (2006)
*Z. usambarensis*	Ext. MeOH (sb)	ENT 30: 5.54 ± 1.70	NF54: 3.20 ± 0.45	97.66 ± 3.6	A.s	17.62	A	Kirira et al. (2006)
*Z. gilletii*	Ext. EtOH (sb)	K1: >5	ND	434.3	L-6	29.9	A/MA/I	Kamanzi Atindehou et al. (2004)
*Z. heterophyllum*	Ext. EtOAc (sb)	ND	3D7: 12.46 ± 4.14	ND	ND	ND	MA	Ledoux et al. (2018)
*Z. djalma-batistae*	Ext. CHCL_3_ (l)	W2: 40.2 ± 3.2	ND	>200	J774	>5.0	MA	Lima (2014)
*Z. djalma-batistae*	Ext. H_2_O (l)	W2: 15.6 ± 2.9	ND	>200	J774	>12.8	MA	Lima (2014)
*Z. djalma-batistae*	Ext. MeOH (l)	W2: >50	ND	ND	ND	ND	MA/I	Lima (2014)
*Z. leprieurii*	Ext. EtOAc (fr)	ND	3D7: >25	ND	ND	ND	MA/I	Tchinda et al. (2009)
Alkaloid								
*Z. chalybeum*	Fagaramide	FCR3: 2.85 ± 1.03	NF54: 16.6 ± 0.50	ND	ND	ND	FCR3: A/NF54: MA	Adia et al. (2016)
*Z. rhoifolium*	Nitidine	*FcB1: 0.80 ± 0.28	*F-32: 0.52 ± 0.1	0.23 ± 0.03	MCF-7	26.3	VA	Bouquet et al. (2012)
*Z. rhoifolium*	Nitidine	*FcM29: 0.49 ± 0.1	ND	8.16 ± 1.04	Vero	0.74	VA	Bouquet et al. (2012)
*Z. chiloperon*	Trans-avicennol	FcB1: 2.2 PF: 1.2 K1: 2.7	F32: 0.5	4.4	MRC5	8.8	A	Cerbrián-Torrejón et al. (2011)
*Z. chiloperon*	Canthin-6-one	FcB1: 4.0 PF: 3.2 K1: 5.3	F32: 2.0	9.4	MRC5	4.7	A	Cerbrián-Torrejón et al. (2011)
*Z. chiloperon*	5-metoxicantina-6-one	K1: 5.1	F32: 10.4	ND	ND	ND	A	Cerbrián-Torrejón et al. (2011)
*Z. heitzii*	Dihydronitidine	ND	3D7: 0.0089 ± 0.0008	ND	ND	ND	VA	Goodman et al. (2016)
*Z. heitzii*	Pellitorine	ND	3D7: 1.96 ± 0.12	ND	ND	ND	A	Goodman et al. (2016)
*Z. heitzii*	Heitziquinone	ND	3D7: 3.55 ± 0.62	ND	ND	ND	A	Goodman et al. (2016)
*Z. heitzii*	Caryophyllene oxide	ND	3D7: >10	ND	ND	ND	MA/I	Goodman et al. (2016)
*Z. heitzii*	Rhoifoline B	ND	3D7: >10	ND	ND	ND	MA/I	Goodman et al. (2016)
*Z. heitzii*	Isoarnottianamide	ND	3D7: >10	ND	ND	ND	MA/I	Goodman et al. (2016)
*Z. zanthoxyloides*	Bis-dihydrocelerythrinyl ether	ND	3D7: 4.3 ± 0.5	ND	ND	ND	A	Goodman et al. (2019)
*Z. zanthoxyloides*	Chelerythrine	ND	3D7: 0.4 ± 0.1	ND	ND	ND	VA	Goodman et al. (2019)
*Z. zanthoxyloides*	γ-fagarin	ND	3D7: 2.2 ± 0.6	ND	ND	ND	A	Goodman et al. (2019)
*Z. zanthoxyloides*	Skimmianine	ND	3D7: 0.7 ± 0.2	ND	ND	ND	VA	Goodman et al. (2019)
*Z. zanthoxyloides*	Pelitorin	ND	3D7: 2.0 ± 0.1	ND	ND	ND	A	Goodman et al. (2019)
*Z. zanthoxyloides*	Buesgenin	ND	3D7: 2.0 ± 0.7	ND	ND	ND	A	Goodman et al. (2019)
*Z. rhoifolium*	Nitidine	FCB1: 1.8	ND	ND	ND	ND	A	Jullian et al. (2006)
*Z. rhoifolium*	Avicine	FCB1: 11.7	ND	ND	ND	ND	MA	Jullian et al. (2006)
*Z. rhoifolium*	Fagaridine	FCB1: 13.6	ND	ND	ND	ND	MA	Jullian et al. (2006)
*Z. rubescens*	N-nornitidine	*FCM29: >64	*N3D7: >64	ND	ND	ND	MA/I	Penali et al. (2007)
*Z. rubescens*	Dimethoxy-2,3 methylenedioxybenzophenanthridine	*FCM2: 92.4 ± 41.9	*3D7: 72.2 ± 13.5	ND	ND	ND	MA	Penali et al. (2007)
*Z. rubescens*	Bis [6-(5,6-dihydrocelerythrinyl ether)	*FCM29: 14.9 ± 1.4	*3D7: 15.3 ± 3.4	ND	ND	ND	MA	Penali et al. (2007)
*Z. rubescens*	Zantomamide	*FCM29: 149.9 ± 59.5	*3D7: 133.8 ± 98.6	ND	ND	ND	I	Penali et al. (2007)
*Z. rubescens*	Lemairamide	*FCM29: 101.1 ± 18.7	*3D7: 89.7 ± 22.7	ND	ND	ND	FCM29: I/3D7: MA	Penali et al. (2007)
*Z. tsihanimposa*	γ-fagarine	*FCM29: 98.4	ND	ND	ND	ND	MA	Randrianarivelojosia et al. (2003)
*Z. tsihanimposa*	N-benzoyltyramine	*FCM29: 165.4	ND	ND	ND	ND	I	Randrianarivelojosia et al. (2003)
*Z. tsihanimposa*	Skimmianine	*FCM29: 134.3	ND	ND	ND	ND	I	Randrianarivelojosia et al. (2003)
*Z. tsihanimposa*	Dictamnine	*FCM29: 332.1	ND	ND	ND	ND	I	Randrianarivelojosia et al. (2003)
*Z. leprieurii*	Tegerrardin A	ND	3D7: 17.3 ± 3.0	ND	ND	ND	MA	Tchinda et al. (2009)
*Z. leprieurii*	Xanthoxoline	ND	3D7: 4.6 ± 0.6	ND	ND	ND	A	Tchinda et al. (2009)
Other classes of isolated metabolites								
*Z. heitzii*	Lignana sesamin	ND	3D7:>10	ND	ND	ND	MA/I	Goodman et al. (2016)
*Z. heitzii*	Terpene isobauerene	ND	3D7: >10	ND	ND	ND	MA/I	Goodman et al. (2016)
*Z. tsihanimposa*	Quinolone 4-methoxy-1–2(1H)quinolinone	*FCM29: 270.7	ND	ND	ND	ND	I	Randrianarivelojosia et al. (2003)
*Z. leprieurii*	Acridone arborinine	ND	3D7: 4.5 ± 1.0	ND	ND	ND	A	Tchinda et al. (2009)
*Z. leprieurii*	Coumarin scoparone	ND	3D7:> 25	ND	ND	ND	MA/I	Tchinda et al. (2009)
*Z. syncarpum*	Amide syncarpamide	*K1: 2.56	*3D7: 3.90	80.66	Vero	22.7	A	Aratikatla et al. (2017)
*Z. leprieurii*	Essential oil (l)	ND	3D7: 62.3 ± 3.4	ND	ND	ND	MA	Tanoh et al. (2020)
*Z. leprieurii*	Essential oil (fr)	ND	3D7: >100	ND	ND	ND	I	Tanoh et al. (2020)
*Z. leprieurii*	Essential oil (sb)	ND	3D7: 36.29 ± 4.2	ND	ND	ND	MA	Tanoh et al. (2020)
*Plasmodium knowlesi*								
*Z. usambarensis*	Ext. H_2_O (sb)	ND	SND: 6.04 ± 0.11	ND	ND	ND	A	Were et al. (2010)
*Z. usambarensis*	Ext. CHCL_3_ (sb)	ND	SND: 26.62 ± 0.09	ND	ND	ND	MA	Were et al. (2010)
*Z. usambarensis*	Ext. EtOAc (sb)	ND	SND: 25.83 ± 0.10	ND	ND	ND	MA	Were et al. (2010)
*Z. usambarensis*	Ext. MeOH (sb)	ND	SND: 48.10 ± 0.07	ND	ND	ND	MA	Were et al. (2010)

AC: activity classification; Ext.: extract; EtOAc: ethyl acetate; H_2_O: water; CH_2_CL_2_:dichloromethane; EtOH: ethanol; MeOH: methanol; PE: petroleum ether; Hex: hexane; CHCL_3_: chloroform; MCW: dichloromethane, methanol, and water; Act: acetone; F: fraction; FA: fraction of alkaloids; b: bark; sb: stem bark; rb: root bark; l: leaves; fr: fruit; ND: not determined; FCR_3_: *P.f.*-resistant strain; W2: *P.f.*-resistant strain; K1: *P.f*.-resistant strain; ENT 30: *P.f.*-resistant strain; FcB1: *P.f.*-resistant strain; PFB: *P.f.*-resistant strain; FcM29: *P.f.*-resistant strain; NF54: *P.f.*-sensitive strain; 3D7: *P.f.*-sensitive strain; F32: *P.f.*-sensitive strain; D6: *P.f.*-sensitive strain; MRA-285 *P.f.*-sensitive strain; F32: *P.f.*-sensitive strain; MRC5: fibroblasts; K562S: chronic myeloid leukemia; A.s.: artemia saline; L-6: rat skeletal myoblasts; J774: murine macrophages; WI-38: normal human fetal lung fibroblasts; Jurkat: leukemia/lymphoma T lymphocytes; Vero: monkey kidney fibroblasts; MCF-7: human breast cancer; MRC5: fibroblasts; SND: strain not determined; I: inactive; MA: moderately active; A: active; VA: very active; * values: µM.

**TABLE 4:** *In vivo* antiparasitic activity against *Plasmodium.*

Vegetal species	Samples	Route of administration	Dose	Parasitemia (%)	Chemosuppression (%)	Inhibition of the parasite load (%)	Protozoan species	Animal species	Author (year)
*Fagara zanthoxyloides*	Ext. MeOH (l)	Oral	200 mg/kg	33	82.37 ± 5.05	68.39 ± 6.07	*P. berghei*	Albino Wistar mice	Enechi et al. (2019)
*F. zanthoxyloides*	Ext. MeOH (l)	Oral	400 mg/kg	41.50	90.75 ± 5.68	84.84 ± 4.86	*P. berghei*	Albino Wistar mice	Enechi et al. (2019)
*F. zanthoxyloides*	Ext. MeOH (l)	Oral	600 mg/kg	44.25	95.95 ± 8.05	92.67 ± 7.41	*P. berghei*	Albino Wistar mice	Enechi et al. (2019)
*Zanthoxylum chalybeum*	Ext. MeOH (l)	Oral	300 mg/kg	37	ND	ND	*P. berghei*	Swiss mice	Muganga et al. (2014)
*Z. chalybeum*	Ext. Ext. H_2_O (r)	Oral	300 mg/kg	35	ND	ND	*P. berghei*	Swiss mice	Muganga et al. (2014)
*Z. chalybeum*	Ext. H_2_O (sb)	Oral	100 mg/kg	17.56 ± 73.62	44.93 ± 11.36	ND	*P. berghei*	Swiss Albino Mice	Musila et al. (2013)
*Z. chalybeum*	Ext. MeOH + CHCL_3_ (sb)	Oral	100 mg/kg	23.09 ± 1.16	27.56 ± 3,635	ND	*P. berghei*	Swiss Albino Mice	Musila et al. (2013)
*Z. rhoifolium Lam*	Ext. H_2_O (b)	Oral	715 mg/kg	ND	ND	78.29	*P. yoelii*	Swiss mice	Bertani et al. (2005)
*Z. usambarense*	Ext. H_2_O (sb)	Intraperitoneal	200 mg/kg	4.64 ± 2.03	64.74	ND	*P. berghei*	BALB/c mice	Were et al. (2010)
*Z. rhoifolium*	Nitidina	Intraperitoneal	10 mg/kg	62.1 ± 8.9	ND	11	*P. vinckei petteri*	Female CD mice (SWISS)	Bouquet et al. (2012)
*Z. rhoifolium*	Nitidina	Intraperitoneal	20 mg/kg	28.5 ± 10.4	ND	59	*P. vinckei petteri*	Female CD mice (SWISS)	Bouquet et al. (2012)

Ext.: extract; H_2_O: water; MeOH: methanol; CHCL_3_: chloroform; l: leaves; r: root; sb: stem bark; s: shell.

**TABLE 5:** *In vitro* antiparasitic activity against the genus *Leishmania*.

Species		Extract, fraction, and isolated substance	Antiparasitic activity (IC_50_ µg/mL ± SD)		Protozoan species	Cytotoxicity (CC_50_ µg/mL ± SD)		Cell line/crustaceans	Selectivity index (SI)	A. C. (Mota et al. 2015)		Author (year)	
			Amastigote	Promastigote									
*Z. armatum*		Ext. EtOH (fr)	ND	21.4 ± 3.3	*Leishmania major*	6.6± 1.1		A.s	ND	A		Alam and Najam us Saqib (2017)	
*Z. juniperinum*		Ext. H_2_O (r)	ND	97.5	*Leishmania* spp.	ND		ND	ND	A		Chinchilla-Carmona et al. (2012)	
*Z. juniperinum*		Ext. EtOH/H_2_O (sb)	ND	23.45	*Leishmania* spp.	ND		ND	ND	A		Chinchilla-Carmona et al. (2012)	
*Z. monophyllum*		Ext. EtOH (b)	ND	17.06 ± 1.49	*L. panamensis*	71.41		HPM	ND	A		Chavez et al. (2014)	
*Z. monophyllum*		Ext. EtOH (b)	ND	25.82 ± 3.15	*L. major*	71.41		HPM	ND	A		Chavez et al. (2014)	
*Z. monophyllum*		FA (b)	ND	77.04 ± 3.72	*L. major*	316.45		HPM	ND	A		Chavez et al. (2014)	
*Z. monophyllum*		FA (b)	ND	61.43 ± 3.05	*L. amazonensis*	316.45		HPM	ND	A		Chavez et al. (2014)	
*Z. rhoifolium*		Ext. EtOH (sb)	ND	88.58	*L. amazonensis*	ND		ND	ND	A		Melo-Neto et al. (2016)	
*Z. rhoifolium*		Ext. EtOH (sb)	ND	16.41	*L. amazonensis*	>400		PCSM	ND	A		Melo-Neto et al. (2016)	
*Z. rhoifolium*		Ext. EtOH (sb)	ND	9.57	*L. amazonensis*	ND		ND	ND	A		Melo-Neto et al. (2016)	
*Z. rhoifolium*		F. Hex (sb)	ND	19.24	*L. amazonensis*	ND		ND	ND	A		Melo-Neto et al. (2016)	
*Z. rhoifolium*		F. Hex (sb)	ND	13.66	*L. amazonensis*	>400		PCSM	ND	A		Melo-Neto et al. (2016)	
*Z. rhoifolium*		F. Hex (sb)	ND	7.96	*L. amazonensis*	ND		ND	ND	A		Melo-Neto et al. (2016)	
*Z. zanthoxyloides*		Ext. CH_2_CL_2_ (sb)	ND	13.5 ± 0.04	*L. donovani*	583.53		Jurkat	ND	A		Dofuor et al. (2019a)	
*Z. zanthoxyloides*		Ext. CH_2_CL_2_ (r)	ND	45.2 ± 0.10	*L. donovani*	247.16		Jurkat	ND	A		Dofuor et al. (2019a)	
*Z. zanthoxyloides*		Ext. CH_2_CL_2_ (l)	ND	>1,000 ± 0.12	*L. donovani*	95.83		Jurkat	ND	I		Dofuor et al. (2019a)	
*Z. armatum*		F. n-hex (fr)	ND	29.6 ± 3.9	*L. major*	6.6± 1.1		A.s	ND	A		Alam and Najam us Saqib (2017)	
Alkaloid													
*Z. rhoifolium*	Nitidine		1.6 ± 0.8 μM	ND	*L. amazonensis*		4.9 µM	M	3.0		A		Castillo et al. (2014)
*Z. rhoifolium*	Avicine		>13.6 μM	ND	*L. amazonensis*		25.6 µM	M	<1.9		A		Castillo et al. (2014)
*Z. rhoifolium*	Fagaridin		>13.6 μM	ND	*L. amazonensis*		19.5 µM	M	<1.4		A		Castillo et al. (2014)
*Z. rhoifolium*	Chelerytrine		0.5 ± 0.0 μM	ND	*L. amazonensis*		3.00 µM	M	6.0		A		Castillo et al. (2014)
*Z. tingoassuiba*	y-fagarine		ND	31.3 ± 1.4 µM	*L. amazonensis*		ND	ND	ND		A		Costa et al. (2018)
*Z. buesgenii*	Buesgenine		5.70 ± 0.41	ND	*L. amazonensis*		43.83	THP-1	7.69		A		Sandjo et al. (2016)
Other classes of isolated metabolites													
*Z. tingoassuiba*	Coumarin 5,7,8-trimethoxycoumarin		ND	57.7 ± 2.2 µM	*L. amazonensis*		ND	ND	ND		A		Costa et al. (2018)
*Z. tingoassuiba*	Coumarin braylin		ND	70.0 ± 1.2 µM	*L. amazonensis*		ND	ND	ND		A		Costa et al. (2018)
*Z. tingoassuiba*	Lignan syringaresinol		ND	12.0 ± 1.2 µM	*L. amazonensis*		ND	ND	ND		A		Costa et al. (2018)

AC: activity classification; Ext.: extract; H_2_O: water; CH_2_CL_2_: dichloromethane; EtOH: ethanol; F.: fraction; n-hex: n-hexane; FA: fraction of alkaloids; Hex: hexane; fr: fruit; r: root; sb: stem bark; s: shell; rb: root bark; l: leaves; A.s: artemia saline; HPM: hamster peritoneal macrophage; PCSM: peritoneal cavity of Swiss mice; Jurkat: leukemia/lymphoma T lymphocytes; M: macrophages; THP-1: human acute monocytic leukemia; A: active; I: inactive; ND: not determined.

**TABLE 6:** *In vivo* antiparasitic activity against *Leishmania.*

Samples	Vegetal species	Route of administration	Dose	Mean parasitemia	Chemosuppression (%)	Inhibition of parasite load (%)	Protozoan species	Animal species	Author (year)
Canthin-6-one	*Zanthoxylum chiloperone*	Oral	10 mg/kg	ND	139.6	171.4	*Leishmania amazonensis*	BALB/c mice	Ferreira et al. (2002)
Canthin-6-one	*Z. chiloperone*	Intralesional	10 mg/kg	ND	15	77.6	*L. amazonensis*	BALB/c mice	Ferreira et al. (2002)
5-methoxycanthin-6-one	*Z. chiloperone*	Oral	10 mg/kg	ND	27.7	68.4	*L. amazonensis*	BALB/c mice	Ferreira et al. (2002)
5-methoxycanthin-6-one	*Z. chiloperone*	Intralesional	10 mg/kg	ND	124.2	21.6	*L. amazonensis*	BALB/c mice	Ferreira et al. (2002)
Avicine	*Z. rhoifolium*	Intralesional	5 mg/kg	10.31×10^6^ ± 0.39	ND	6.2	*L. amazonensis*	BALB/c mice	Castillo et al. (2014)
Fagaridin	*Z. rhoifolium*	Intralesional	5 mg/kg	3.96×10^6^ ± 0.48	ND	59.6	*L. amazonensis*	BALB/c mice	Castillo et al. (2014)
Chelerytrine	*Z. rhoifolium*	Intralesional	5 mg/kg	6.75×10^6^ ± 0.29	ND	29	*L. amazonensis*	BALB/c mice	Castillo et al. (2014)

ND, not determined.

**TABLE 7:** *In vitro* antiparasitic activity against *Trypanosoma cruzi.*

Species	Extract, fraction, and isolated substance	Antiparasitic activity (IC_50_ µg/mL ± SD)		Cepa	Cytotoxicity (CC_50_ µg/mL ± SD)	Cell line/crustaceans	Selectivity index (SI)	A.C	Author (year)
		Epimastigote	Trypomastigote						
*Z. naranjillo*	Ext. Hex (b)	ND	1.53	Y	ND	ND	ND	GA	Bastos et al. (1999)
*Z. naranjillo*	Ext. Hex (b)	ND	2.00	Bolivia	ND	ND	ND	GA	Bastos et al. (1999)
*Z. monogynum*	Ext. EtOH (l)	69.03 ± 0.34	ND	Y	444	RAW 264.7	ND	LA	Da Silva et al. (2019)
*Z. monogynum*	Ext. Hex (l)	73.95 ± 0.06	ND	Y	ND	ND	ND	LA	Da Silva et al. (2019)
*Z. monogynum*	Ext. CH_2_CL_2_ (l)	78.31 ± 0.14	ND	Y	ND	ND	ND	LA	Da Silva et al. (2019)
*Z. minutiflorum*	Ext. CH_2_CL_2_ (b)	ND	2.13	ND	ND	ND	ND	GA	Mafezoli et al. (2000)
*Z. minutiflorum*	Ext. MeOH (b)	ND	4.84	ND	MD	ND	ND	GA	Mafezoli et al. (2000)
*Z. minutiflorum*	Ext. EtOH (l)	88.75 ± 0.47	ND	Dm28c	5.75	RAW 264.7	ND	LA	Da Silva et al. (2019)
*Z. minutiflorum*	Ext. Hex (l)	35.53 ± 0.21	ND	Dm28c	ND	ND	ND	MA	Da Silva et al. (2019)
*Z. minutiflorum*	Ext. CH_2_CL_2_ (l)	90.52 ± 1.18	ND	Dm28c	ND	ND	ND	LA	Da Silva et al. (2019)
*Z. minutiflorum*	F. Hex (b)	ND	1.86	Y	ND	ND	ND	GA	Mafezoli et al. (2000)
*Z. minutiflorum*	F. EtOAc (b)	ND	3.61	Y	ND	ND	ND	GA	Mafezoli et al. (2000)
*Z. minutiflorum*	F. But (b)	ND	2.84	Y	ND	ND	ND	GA	Mafezoli et al. (2000)
*Z. tingoassuiba*	Coumarin 5,7,8-trimethoxycoumarin	25.5	ND	Y	ND	ND	ND	MA	Costa et al. (2018)
*Z. tingoassuiba*	Coumarin braylin	59.8	ND	Y	ND	ND	ND	LA	Costa et al. (2018)
*Z. Tingoassuiba*	Lignan syringoresinol	7.6	ND	Y	ND	ND	ND	GA	Costa et al. (2018)
*Z. tingoassuiba*	Alkaloid y-fagarine	33.4	ND	Y	ND	ND	ND	MA	Costa et al. (2018)

AC: activity classification; Ext.: extract; Hex: hexane; EtOH: ethanol; CH_2_CL_2_: dichloromethane; MeOH: methanol; F.: fraction; EtOAc: ethyl acetate; But: butanol; b: bark; l: leaves; Dm28c: resistant strain of *Trypanosoma cruzi*; Y: sensitive strain of *T. cruzi*; Bolivia: sensitive strain of *T. cruzi*; RAW 264.7: murine macrophages; GA: good active; MA: moderately active; LA: low active; ND: not determined.

**TABLE 8:** *In vivo* antiparasitic activity against *Trypanosoma cruzi*.

Vegetal species	Samples	Route of administration	Dose	Parasitemia mean	Protozoan species	Animal species	Author (year)
*Zanthoxylum chiloperone*	Ext. bruto (b)	Oral	50 mg/kg	0.7×10^4^ ± 0.8	*Trypanosoma cruzi*	BALB/c mice	Ferreira et al. (2007)
*Z. chiloperone*	Ext. bruto (b)	Subcutaneous	50 mg/kg	2.7×10^4^ ± 1.7	*T. cruzi*	BALB/c mice	Ferreira et al. (2007)
*Z. chiloperone*	Ext. EtOH (l)	Oral	10 mg/kg	2 × 10^5^	*T. cruzi* - Cepa CL	BALB/c mice	Ferreira et al. (2011)
*Z. chiloperone*	Ext. EtOH (l)	Subcutaneous	10 mg/kg	4 × 10^5^	*T. cruzi* - Cepa CL	BALB/c mice	Ferreira et al. (2011)
*Z. chiloperone*	Canthin-6-one	Oral	5 mg/kg	0.1×10^4^ ± 0.2	*T. cruzi*	BALB/c mice	Ferreira et al. (2007)
*Z. chiloperone*	Canthin-6-one	Subcutaneous	5 mg/kg	3.3×10^4^ ± 2.7	*T. cruzi*	BALB/c mice	Ferreira et al. (2007)
*Z. chiloperone*	5-methoxycanthin-6–1	Oral	5 mg/kg	49.3×10^4^ ± 27.8	*T. cruzi*	BALB/c mice	Ferreira et al. (2007)
*Z. chiloperone*	5-methoxycanthin-6–1	Subcutaneous	5 mg/kg	28.4×10^4^ ± 23.7	*T. cruzi*	BALB/c mice	Ferreira et al. (2007)
*Zanthoxylum chiloperone*	Canthin-6-one N-oxide	Oral	5 mg/kg	0	*T. cruzi*	BALB/c mice	Ferreira et al. (2007)

Ext.: extract; EtOH: ethanol; b: bark; l: leaves.

**TABLE 9:** *In vitro* antiparasitic activity against *Trypanosoma brucei*.

Species	Extract, fraction, and isolated substance	Protozoan activity (IC_50_ µg/mL ± SD)	Cytotoxicity (CC_50_ µg/mL ± SD)	Cell line/crustaceans	Selectivity index (SI)	A. C	Author (year)
*Trypanosoma brucei*							
*Z. zanthoxyloides*	Ext. CH_2_CL_2_ (r)	3.41	ND	ND	ND	GA	Dofuor et al. (2019a)
*Z. zanthoxyloides*	Ext. CH_2_CL_2_ (sb)	39.43 ± 0.04	ND	ND	ND	MA	Dofuor et al. (2019a)
*Z. zanthoxyloides*	Ext. CH_2_CL_2_ (l)	27.73	ND	ND	ND	MA	Dofuor et al. (2019a)
*Z. zantoxiloides*	Ext. EtOH (r)	39.43	95.83	Jurkat	6.27	MA	Ohashi et al. (2018)
*Z. zantoxiloides*	Ext. EtOH (sb)	5.96	95.83	Jurkat	97.91	GA	Ohashi et al. (2018)
*Z. zantoxiloides*	Ext. EtOH (l)	27.73	95.83	Jurkat	3.46	MA	Ohashi et al. (2018)
*Z. zanthoxyloides*	Alquilamida tortozanthoxilamida	7.78	214.96	RAW	26.62	GA	Dofuor et al. (2019b)
*Z. xanthoxyloides*	Ext. CH_2_CL_2_ (sb)	<8.6	56	WI-38	1.2	GA	Freiburghaus et al. (1996)
*Z. xanthoxyloides*	Ext. P.E (sb)	<5.6	56	WI-38	1.2	GA	Freiburghaus et al. (1996)
*Z. xanthoxyloides*	Ext. MeOH (sb)	7.4	19	WI-38	5.9	GA	Freiburghaus et al. (1996)
*Z. chalybeum*	Ext. MeOH (l)	36.00	137.31	HL-60	3.81	MA	Nibret et al. (2010)
*Z. chalybeum*	Ext. CH_2_CL_2_ (l)	11.02	30.16	HL-60	2.74	MA	Nibret et al. (2010)
*Z. gilletii*	Ext. EtOH (sb)	14.5	434.3	L-6	29.9	MA	Kamanzi Atindehou et al. (2004)

AC: activity classification; Ext.: extract; CH_2_CL_2_: dichloromethane; PE: petroleum ether; MeOH: methanol; EtOH: ethanol; r: root; sb: stem bark; l: leaves; WI-38: human fetal lung diploid cells; HL-60: human leukemia cells; Jurkat: leukemia/lymphoma T lymphocytes; L-6: rat skeletal myoblasts; RAW: rat macrophages; GA: good active; MA: moderately active; ND: not determined.

### 3.4 *Plasmodium*

One study evaluated the antiplasmodic activity against 12 alkaloid fractions from the bark of the *Z. zanthoxyloides* stem against resistant strains of *P. falciparum*. Fractions 1–5, 10, and 12 were active and non-cytotoxic to chronic myeloid leukemia strain (Table 3; Gansane et al., 2010). The ethyl acetate extract from the stem bark of *Zanthoxylum chalybeum* was active against sensitive and resistant strains *of P. falciparum* (Table 3; Adia et al., 2016).

Ethanolic ethyl acetate and aqueous extracts from the *Z. chalybeum* root bark showed activity against the same resistant strains (Table 3; Gessler et al., 1994). In addition, the aqueous extract from the *Z. chalybeum* root bark was active against resistant strains of the parasite, and the methanol extract was active against resistant and sensitive strains (Table 3; Muthaura et al., 2015).

From *Z. rhoifolium*, one alkaloid was isolated, and the alkaloid nitidine was considered active against the resistant strain of the *P. falciparum* (Table 3; Jullian et al., 2006). The alkaloid nitidine isolated from *Z. rhoifolium* was active against two resistant strains and a sensitive strain of *P. falciparum*, and it was not cytotoxic against monkey kidney fibroblast cells (Table 3; Bouquet et al., 2012).

The hexane extract from the bark of *Z. heitzii* was active on sensitive strains of *P. falciparum.* The alkaloids dihydronitidine, pellitorine, heitziquinone, caryophyllene oxide, rhoifoline B, and isoarnottianamide were isolated from the same species. From them, dihydronitidine and pellitorine showed activity against *P. falciparum* strains.

Finally, for *in vitro* studies, the syncarpamide, isolated from *Z. syncarpum*, was active in resistant and sensitive strains of *P. falciparum*, being non-cytotoxic against the normal monkey kidney fibroblast cells with high SI (Table 3; Aratikatla et al., 2017).

Regarding *in vivo* studies, percentage parasitemia and percentage chemosuppression were determined using the formula % parasitemia = (total number of parasite cells/total number of cells) x 100% and chemosuppression = [(negative control parasitemia) − (parasitemia with drug)]/negative control parasitemia (Hilou et al., 2006). After oral treatment with 200 mg/kg of methanol extract from *Fagara zanthoxyloides* in albino Wistar mice infected with *Plasmodium berghei*, the level of parasitemia was 33%, chemosuppression was 82.37%, and the inhibition rate of the parasite load was 68.39%. After treatment with a dose of 400 mg/kg of the same extract, the level of parasitemia was 41.50%, chemosuppression was 90.75%, and the inhibition rate of the parasite load was 84.84%. After treatment with the highest dose (600 mg/kg) of the extract, the parasitemia was 44.15%, with a chemosuppression of 95.95%, and inhibition of the parasite load of 92.67%. Furthermore, the non-lethality of the extract was confirmed, even at the highest dosage (5,000 mg/kg), having a CC_50_ of 28.21 ± 1.30 μg/ml (Table 4; Enechi et al., 2019).

An oral treatment with 100 mg/kg of aqueous and methanol + chloroform extract obtained from the bark of the *Zanthoxylum chalybeum* stem was administered to Swiss albino mice infected with *Plasmodium berghei*; parasitemia of 17.56% and chemosuppression of 44.93% were found after treatment, and parasitemia of 23.09% and chemosuppression of 27.56% after treatment with methanol + chloroform extract were also found. The two extracts were not cytotoxic (CC_50_: 268.28 and 25.78 μg/ml, respectively) at a dosage >1,000 μg/ml (Table 4), and for each specific blood smear for a given mouse, four magnification fields were observed, and the number of parasitized cells and the total number of cells in the magnification field were recorded. The data obtained were used to determine the percentage parasitemia and percentage chemosuppression in each mouse (Musila et al., 2013).

Finally, the inhibition of the parasite load in Swiss mice infected with *P. yoelii* after oral treatment with 715 mg/kg of the aqueous extract from *Z. rhoifolium* was 78.29% (Table 4; Bertani et al., 2005). In this study, there was no calculation of parasitemia.

### 3.5 *Leishmania*

In *in vitro* studies, the ethanolic extract and the hexane fraction from the bark of the *Z. rhoifolium* stem showed activity against *Leishmania amazonensis* promastigotes in 24, 48, and 72 h of treatment (Table 5; Melo-Neto et al., 2016). In the same study, the alkaloids chelerythrin and nitidine, isolated from *Z. rhoifolium*, showed activity against *L. amazonensis* amastigotes (Table 5; Castillo et al., 2014).

The dichloromethane extract from the bark of the *Z. zanthoxyloides* stem showed activity against *L. donovani* promastigotes; it was not cytotoxic in Jurkat cells (Table 5; Dofuor et al., 2019a).

The ethanolic extract and the alkaloid fraction from the bark of *Z. monophyllum* showed activity against *L. major* and *L. panamensis* promastigotes (Table 5; Enciso et al., 2014). Moreover, the ethanolic extract and the n-hexane fraction from *Z. armatum* fruits showed activity when tested against *Leishmania major* promastigotes (Table 5; Alam and Najam us Saqib, 2017).

The hydroethanolic and the aqueous extracts from the bark of *Z. juniperinum* showed activity against the promastigote form of *Leishmania* spp. (Table 5; CHINCHILLA-(Chinchilla-Carmona et al., 2012). In addition, the alkaloid buesgenine isolated from *Z. buesgenii* showed activity against amastigotes of *L. amazonensis* (Table 5; Sandjo et al., 2016). Also, the syringoresinol lignan, isolated from *Z. tingoassuiba*, was active against *L. amazonensis* promastigotes (Table 5; Costa et al., 2018).

Regarding *in vivo* studies, only isolated compounds of the genus were tested against *Leishmania*. The antileishmanial activity of canthin-6-one and 5-methoxycanthin-6-one isolated from *Zanthoxylum chiloperone* was evaluated in BALB/c mice infected with *Leishmania amazonensis* strains at a dose of 10 mg/kg by different routes (oral and intralesional). After oral treatment with canthin-6-one and 5-methoxycanthin-6-one, the parasite load inhibition rate was 171.4% and 68.4%, respectively, and chemosuppression of 139.6% and 27.7%, respectively. After treatment *via* the intralesional route, the parasitic inhibition rate was 77.6% and 21.6%, respectively, and the level of chemosuppression was 15% and 124.2%. It did not present toxicity, and its lethal dose was >400 mg/kg intraperitoneally (Table 6; Ferreira et al., 2002).

The alkaloids fagaridin and chelerythrine isolated from *Zanthoxylum rhoifolium* were also evaluated to assess their antileishmanial activity in BALB/c mice infected with *L. amazonensis* strains treated with a dose of 5 mg/kg by intralesional injection. After treatment, the parasitemia was 3.96×10^6^ ± 0.48 for fagaridin and 6.75×10^6^ ± 0.29 for chelerythrine; the inhibition rate of the parasite load was 59.6% and 29%, respectively (Table 6; Castillo et al., 2014). In none of the *in vivo* studies was there an analysis of parasitemia in percentage (%), only the mean was presented.

### 3.6 *Trypanosoma cruzi*

The hexane extract from the bark of *Zanthoxylum naranjillo* showed high activity against different strains of *Trypanosoma cruzi* trypomastigotes (Table 7; Bastos et al., 1999), followed by the hexane fraction of *Z. minutiflorum* with activity against *T. cruzi* trypomastigotes (Table 7; Mafezoli et al., 2000). The lignan compound syringoresinol isolated from the species *Z. tingoassuiba* was the isolated compound that showed the strongest activity against *T. cruzi* epimastigotes (Table 7; Costa et al., 2018). No studies examined compound activity against cells in the amastigote phase.

In *in vivo* studies, the crude extract of the stem of *Zanthoxylum chiloperone* showed the strongest antitrypanosome activity, being evaluated at a dose of 50 mg/kg orally and subcutaneously in BALB/c mice infected with *Trypanosoma cruzi*. After oral treatment, a parasitemia of 0.7×10^4^ ± 0.8 was found, and a parasitemia of 2.7×10^4^ ± 1.7 was found after subcutaneous administration (Table 8; Ferreira et al., 2007).

In addition, the alkaloid canthin-6-one, also isolated from the species *Zanthoxylum chiloperone*, was tested in BALB/c mice infected with *T. cruzi* strains at a dose of 5 mg/kg, orally and subcutaneously. After exposure to oral treatment, the parasitemia was 0.1×10^4^ ± 0.2, and after subcutaneous treatment, the parasitemia was 3.3×10^4^ ± 2.7, representing the compound with the strongest *in vivo* activity (Table 8; Ferreira et al., 2007).

### 3.7 *Trypanosoma brucei*

The dichloromethane extract obtained from the root of *Z. zanthoxyloides* showed the strongest activity against *T. brucei* (Table 9; Dofuor et al., 2019a). Moreover, the alkylamide tortozanthoxylamide, isolated from *Z. zanthoxyloides*, was the only isolated compound of the genus that was found in tests against *T. brucei* showing activity against the protozoan, moderate cytotoxicity in rat macrophages, and high SI (Table 9; Dofuor et al., 2019b).

## 4 Discussion

This systematic review provides information on four types of diseases caused by protozoa and the therapeutic potential of species from the genus *Zanthoxylum*. Original articles reporting data on preclinical experiments (*in vitro* and/or *in vivo*) with extracts, fractions, and isolated compounds of the genus *Zanthoxylum* against protozoa were included in the review. Two instruments were used to minimize the risk of bias: the guideline, developed by Faggion et al. (2012) for pre-clinical *in vitro* studies, and SYRCLE, developed by Hooijmans et al. (2014) to evaluate the *in vivo* studies. It is noteworthy that the analysis was performed by two independent evaluators, with the addition of a third when necessary, ensuring robustness and veracity of the analyzed data.

The treatment for diseases caused by protozoa, such as malaria, leishmaniasis, Chagas disease, and sleeping sickness, has several adverse effects, high toxicity, parasite resistance, and a reduced number of antiprotozoal drugs (Ohashi et al., 2018). Therefore, it is essential to search for new therapeutic alternatives from other sources, such as medicinal plants. Thus, we emphasize the importance of this review as a beginning in the analysis of possible promising species for the treatment of the diseases under study.

The lack of standard protocols for the evaluation of *in vitro* and *in vivo* results made it difficult to interpret the results; however, to minimize such factors, the activity classification the parameters of Dolabela et al. (2015) for the genus *Plasmodium* and those of Mota et al. (2015) for the genus *Leishmania* were used, and the following parameters were adopted to evaluate the activity against *Trypanosoma cruzi* and *Trypanosoma brucei*: IC_50_ < 10 μg/ml, good activity; IC_50_ of 10–50 μg/ml, moderate activity; IC_50_ 50–100 μg/ml, low activity; and IC_50_ > 100 μg/ml, inactive.

Initially, the *in vitro* and *in vivo* antimalarial activity of *Zanthoxylum* was evaluated. Only 10 species were evaluated *in vitro*, using sensitive and resistant clones of *Plasmodium falciparum*. From the species, the *Z*. *zanthoxyloides* was widely studied *in vitro*, having the activity of its extracts, fractions, and isolated substances evaluated (Gansane et al., 2010; Goodman et al., 2019). Extracts obtained from this species were active in a chloroquine-resistant clone of *P. falciparum*, while the fractions were promising in a sensitive clone (Table 3). Also, extracts from the bark of this species were promising against *L. donovani* (Table 5).

The species *Z. zanthoxyloides* stood out due to its activity against African trypanosomiasis (sleeping sickness) caused by *T. brucei*. The dichloromethane extract from this species was the most promising (Dofuor et al., 2019a), followed by petroleum ether and methanol extracts (Dofuor et al., 2019a). From these, methanol extract showed less cytotoxic effect with a good selectivity index (Freiburghaus et al., 1996). In the study by Dofuor et al. (2019a), the dichloromethane extract presented aromatic hydrocarbons as its main constituents, indicative of metabolites such as terpenes. Thus, it suggested terpenes may be responsible for the antitrypanosomal activity.

All alkaloids isolated from the species were promising as antimalarials (Table 3). The likely mechanism of action of the alkaloids is explained by their ability to form complexes with the heme group and inhibit the formation of b-hematin (O'Neill et al., 2009; Kelly et al., 2009; Bouquet et al., 2012). The fagaronin inhibits topoisomerases I and II and acts as a DNA-intercalating agent (Larsen et al., 1993), and berberine inhibits the *Plasmodium* telomerase (Sriwilaijareon et al., 2002; Parida et al., 2014). The alkaloid flavopereirin also was active against *L. amazonensis* promastigotes, and this activity is attributable to oligopeptidase binding (Silva e Silva et al., 2020).

Other alkaloids have already been subjected to molecular docking studies to suggest possible therapeutic targets against parasites, such as cassin and (-)-3-O- acetylspectaline, which bind to the arginase in *L. amazonensis*, being embedded in the binding site of the enzyme, with hydrophobic bonds forming the ligand–arginase complex, which may explain the leishmanicidal profile of the compounds shown in the studies (Lacerda et al., 2018). This is one of the possible mechanisms of action of this class of substances.

From *Z. chiloperone*, the alkaloid canthin-6-one was isolated. This alkaloid showed antimicrobial (Thouvenel et al., 2003) and leishmanicidal (Ferreira et al., 2002) activities and was active against *T. cruzi* (Ferreira et al., 2007). However, the mechanism of action of canthin-6-one is entirely unknown, but its trypanocidal activity may suggest, as a first hypothesis, the inhibition of the sterol 14α-demethylase in *T. cruzi* intracellular amastigotes (Ferreira et al., 2007), a mechanism similar to that proposed for triazole antifungals (Molina et al., 2000; Lira et al., 2001; Urbina et al., 2003). This alkaloid also showed promising activity against *L. amazonenzis* (Ferreira et al., 2002), making it urgent to investigate its possible mechanism of action.

The alkaloids from the benzophenanthridine class (chelerythrine, skimmianine, and buesgenin) were also active against chloroquine-sensitive strains (Table 3; Goodman et al., 2019), with anti-inflammatory (Chaturvedi et al., 1997; Dvořák et al., 2006) and antimicrobial (Croaker et al., 2016) activities. An *in vivo* study using methanol extract of *Z. zanthoxyloides* administered orally in Wistar albino mice was found. This sample showed chemosuppression and inhibition of the parasite load proportional to dosage (Table 4; Enechi et al., 2019), the antimalarial mechanism of action being explained by the presence of fagaronine and berberine in its extract (Elujoba et al., 2005).

The alkylamide tortozanthoxylamide isolated from *Z. zanthoxyloides* showed promising results against *T. brucei* and a good selectivity index (Dofuor et al., 2019b). Some studies suggest the activity of this compound may occur by targeting the functioning of the parasite’s cell cycle through the inhibition of DNA synthesis, which is replicated in the S phase of parasite reproduction, and inhibiting the karyokinesis processes and parasite cytokinesis (Dofuor et al., 2019b). In summary, the antiparasitic activity of this genus is related to the alkaloids; however, preliminary results suggest that different signaling pathways may be involved in the activities (Dofuor et al., 2019b).

The extracts from *Z. rhoifolium* presented the best *in vitro* activity against strains resistant to CQ, and fractions obtained from these extracts were active in sensitive clones. From the isolated compounds of the genus, in *in vitro* studies, the alkaloid chelerythrine, isolated from *Z. rhoifolium*, showed the strongest activity. The compound showed good *in vivo* activity in parasitemia indices, and it was the least toxic compound (Castillo et al., 2014). The alkaloid nitidine isolated from this species obtained IC_50_ < 1 μg/ml in a resistant clone and a satisfactory selectivity index (>10) when compared to a non-tumor cell line (Table 3; Bouquet et al., 2012), being described as an active antimalarial principle (Bai et al., 2006; Nyangulu et al., 2010; BOUQUET et al., 2012).

Also, it showed anti-leishmania activity *in vitro*, with the hexane fraction (apolar), rich in terpenes, being the most promising (Melo-Neto et al., 2016), and the following compounds were isolated from this fraction: 7-O-2-quinolone ether; (13S)-labdane-8α, 15-diol; (13R)-labdane-8α; 15-diol; and 13-(S)-8α-13-epoxylabd-14-ene (Santiago-Brugnoli et al., 2013). The leishmanicidal activity of terpenes has been described in different studies (Cechinel-Filho and Yunes, 1998; Arruda et al., 2005), as having possible direct mechanisms of action in the inhibition of protease activity, lipid synthesis, cell cycle, or indirectly through modulation of macrophage activation (Soares et al., 2012). Metabolites as the nerolidol, an oxygenated sesquiterpene, effectively inhibit the biosynthesis of isoprenoids such as dolicho, ergosterol, and ubiquinone in promastigotes (Arruda et al., 2005; Rodrigues et al., 2013). Two terpenes isolated from *C. cajucara*, namely, t-dehydrocrotonin and t-crotonin, inhibited trypanothione reductase in *L. amazonensis* promastigotes (Lima, 2014).

Among the analyzed species, the *Z. chalybeum* stood out due to the activity of its ethyl acetate extract and the alkaloid fagaramide against sensitive and resistant strains of *Plasmodium* (Adia et al., 2016). Also, from this species, two extracts (methanol + aqueous extract and methanol + chloroform + aqueous extract) decreased parasitemia and promoted high chemosuppression in Swiss mice infected with *Plasmodium berghei* (Musila et al., 2013; Mugunga et al., 2014). Considering that phytochemical analyses of this species revealed several alkaloid substances, such as benzophenanthridine, chelerythrine, nitidine, and 8-O-demethylchelerythrine (Kato et al., 1996; Tian et al., 2017), it is suggested that its activity against *Plasmodium* results from the blocking or depolymerization of the cytoskeleton in red blood cells to prevent the entry of the protozoan (Fernandes, 2017).

The species *Z. heitzii* also had good activity against sensitive *Plasmodium* strains. Their study reports the isolation of some classes of substances; however, only the alkaloids didydronitidine and heitziquinone showed activity against the tested strains. The study suggested that the activity of the alkaloid dihydronitidine is equal to that of “delayed death” drugs (Goodman et al., 2016), common to compounds that target the parasite’s epicoplast (Goodman et al., 2007). However, the alkaloid heitziquinone comes from benzo(c)phenanthridine, and previous studies found that this class is very sensitive to small changes in chemical structure (Nyangulu et al., 2010), and its structure–activity mechanism comes from an open C-ring (Goodman et al., 2016); this can cause difficulty to fully understand its antiplasmodial mechanism.

In the compilation of studies of the genus *Zanthoxylum* against *T. cruzi*, some *in vitro* analyses were highlighted. The hexane extract from *Z. naranjillo* against the *T. cruzi* trypomastigote form presented good activity in both tested strains (sensitive and resistant; Bastos et al., 1999); however, this is the only study that affirms the trypanocidal activity of the species. Complementary studies to identify the metabolite responsible for the activity and the mechanism are important.

The dichloromethane and methanol extracts and ethyl acetate, hexane, and butanol fractions from *Z. minutiflorum* showed strong activity against the trypomastigote form (Mafezoli et al., 2000). The *in vivo* trypanocidal action has also been reported in the literature through an index of published abstracts (Ferreira et al., 2003); however, this was the only *in vivo* study for this species reported in the literature.

The trypanocidal activity may be related to different chemical constituents of plants, such as isoquinoline alkaloids, lignans, coumarins, flavonoids, and terpenes (Prieto et al., 2011). In the *in vitro* study of the *Z. tingoassuiba*, the isolated compound syringoresinol, a furofuran lignan, showed activity against the epimastigote form of the Y strain, and its isolation from this plant species was reported for the first time in the literature (Costa et al., 2018). Through molecular docking studies, it is suggested that the likely mechanism of action of lignans occurs by interrupting the divisions and other cellular functions of the parasite, with tubulin being a possible biological target (Corrêa, 2015).

## 5 Conclusion

**FIGURE 2:**
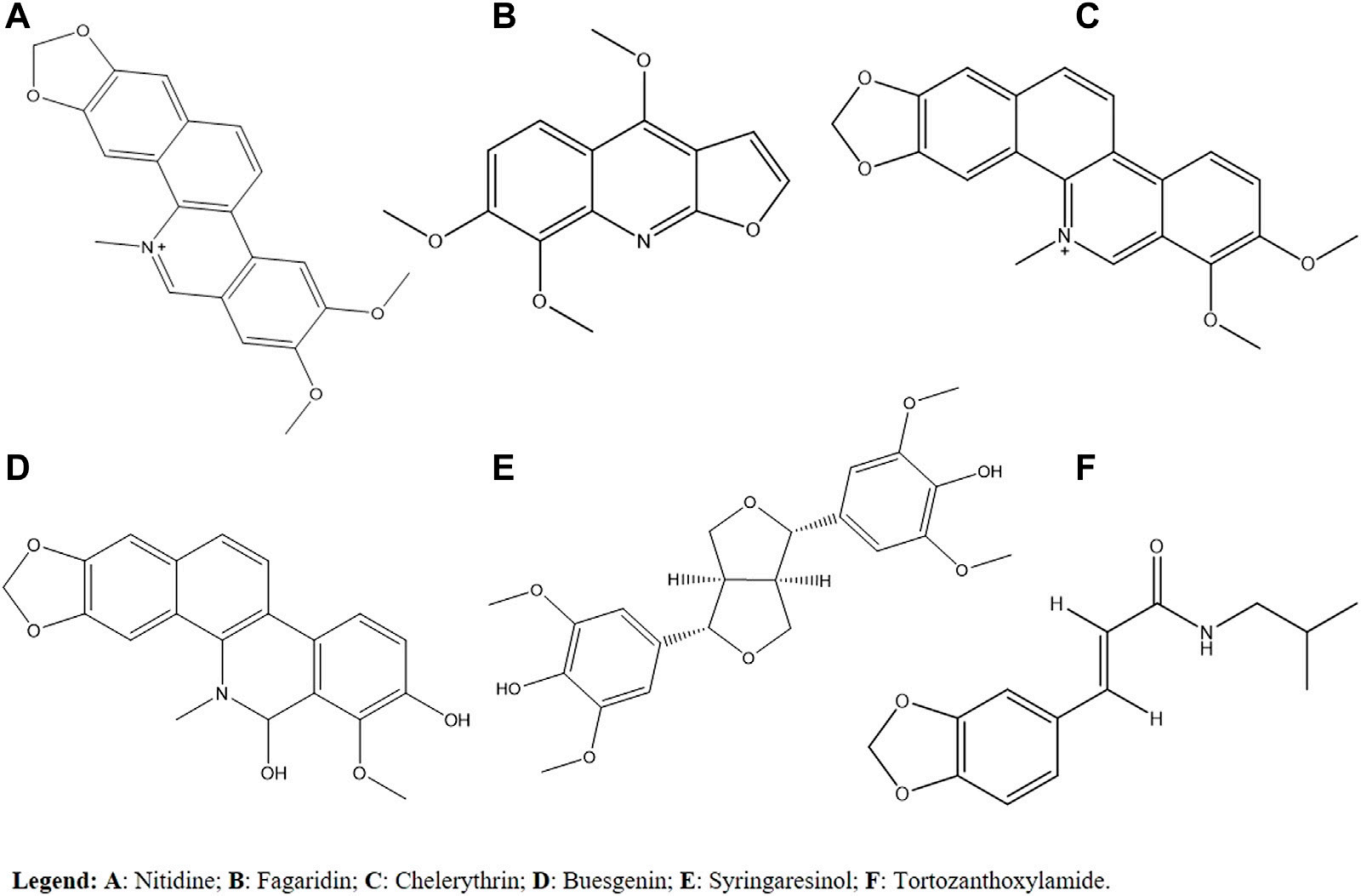
Most promising secondary metabolites for the diseases studied.

Isolated fractions and substances from different *Zanthoxylum* species seem to be promising as sources of active molecules in disease-causing parasites. When considering the cytotoxicity and antimalarial activity *in vitro* and *in vivo*, *Z. rhoifolium* is the most promising species due to metabolites such as alkaloids in its composition. When considering the antileishmanial activity, it is suggested that alkaloids and terpenes are responsible for the activity, mainly on the *Z. rhoifolium*. For Chagas disease, in the *in vitro* analysis, the most promising species was *Z. minutiflorum* due to its activity against trypomastigotes. As for *in vivo* analysis, the most promising species was *Z. chiloperone*, based on alkaloids in its composition. Finally, for sleeping sickness, the most promising species *in vitro* studies was *Z. zanthoxyloides*, and its activity may be related to metabolites such as terpenes and tortozanthoxylamide. In general, the most promising metabolites for the studied diseases are described in Figure 2. So far, there are no studies on the *in vivo* infection of the disease. The importance of toxicity studies in animals and the evaluation of the genotoxic, mutagenic, and carcinogenic potential of the species are highlighted.

## Data Availability

The original contributions presented in the study are included in the article/Supplementary Material; further inquiries can be directed to the corresponding author.

## Glossary

AC: activity classification
Bolivia: sensitive strain of *T. cruzi*
DeCs: descriptors in health sciences
NDs: neglected diseases
NTDs: neglected tropical diseases
BVS: Virtual Health Library–biblioteca virtual da saúde
Mesh: medical subjects
N: not relevant to the item
S: showed relevance to the item
M: presented medium relevance to the item
IC_50_: 50% inhibitory concentration
CC_50_: 50% cytotoxic concentration
SI: selectivity index
Ext: extract
EtOAc: ethyl acetate
H_2_O: water
CH_2_CL_2_: dichloromethane
EtOH: ethanol
MeOH: methanol
PE: petroleum ether
Hex: hexane
CHCL_3_: chloroform
MCW: dichloromethane, methanol, and water
Act: acetone
lr: root
s: shell
n-hex: n-hexane
F: fraction
FA: fraction of alkaloids
b: bark
sb: stem bark
rb: root bark
l: leaves
fr: fruit
ND: not determined
SND: strain not determined
I: inactive
MA: moderately active
A: active
VA: very active
GA: good active
LA: low active
FCR3: *Plasmodium falciparum*-resistant strain
W2: *Plasmodium falciparum*-resistant strain
K1: *Plasmodium falciparum*-resistant strain
ENT 30: *Plasmodium falciparum*-resistant strain
FcB1: *Plasmodium falciparum*-resistant strain
PFB: *Plasmodium falciparum*-resistant strain
FcM29: *Plasmodium falciparum*-resistant strain
NF54: *Plasmodium falciparum*-sensitive strain
3D7: *Plasmodium falciparum*-sensitive strain
F32: *Plasmodium falciparum*-sensitive strain
D6: *Plasmodium falciparum*-sensitive strain
MRA-285: *Plasmodium falciparum*-sensitive strain
MRC5: fibroblasts
K562S: chronic myeloid leukemia
A.s.: artemia saline
L-6: rat skeletal myoblasts
J774: murine macrophages
Jurkat: leukemia/lymphoma T lymphocytes
VERO: monkey kidney fibroblasts
MCF-7: human breast cancer
HPM: hamster peritoneal macrophage
BALB/c: Albino mice c genetic line
PCSM: peritoneal cavity of Swiss mice
M: macrophages
THP-1: human acute monocytic leukemia
But: butanol
Dm28c: resistant strain of *T. cruzi*
Y: sensitive strain of *T. cruzi*
RAW 264.7: murine macrophages
WI-38: diploid human cell line composed of fibroblasts from lung tissue
HL-60: human leukemia cells
RAW: rat macrophages
