# Supplementary figures and images for: Activity of the genus Zanthoxylum against diseases caused by protozoa: A systematic review
Source: Front Pharmacol. 2023 Jan 9;13:873208. doi: 10.3389/fphar.2022.873208 (PMC9868958; doi:10.3389/fphar.2022.873208)

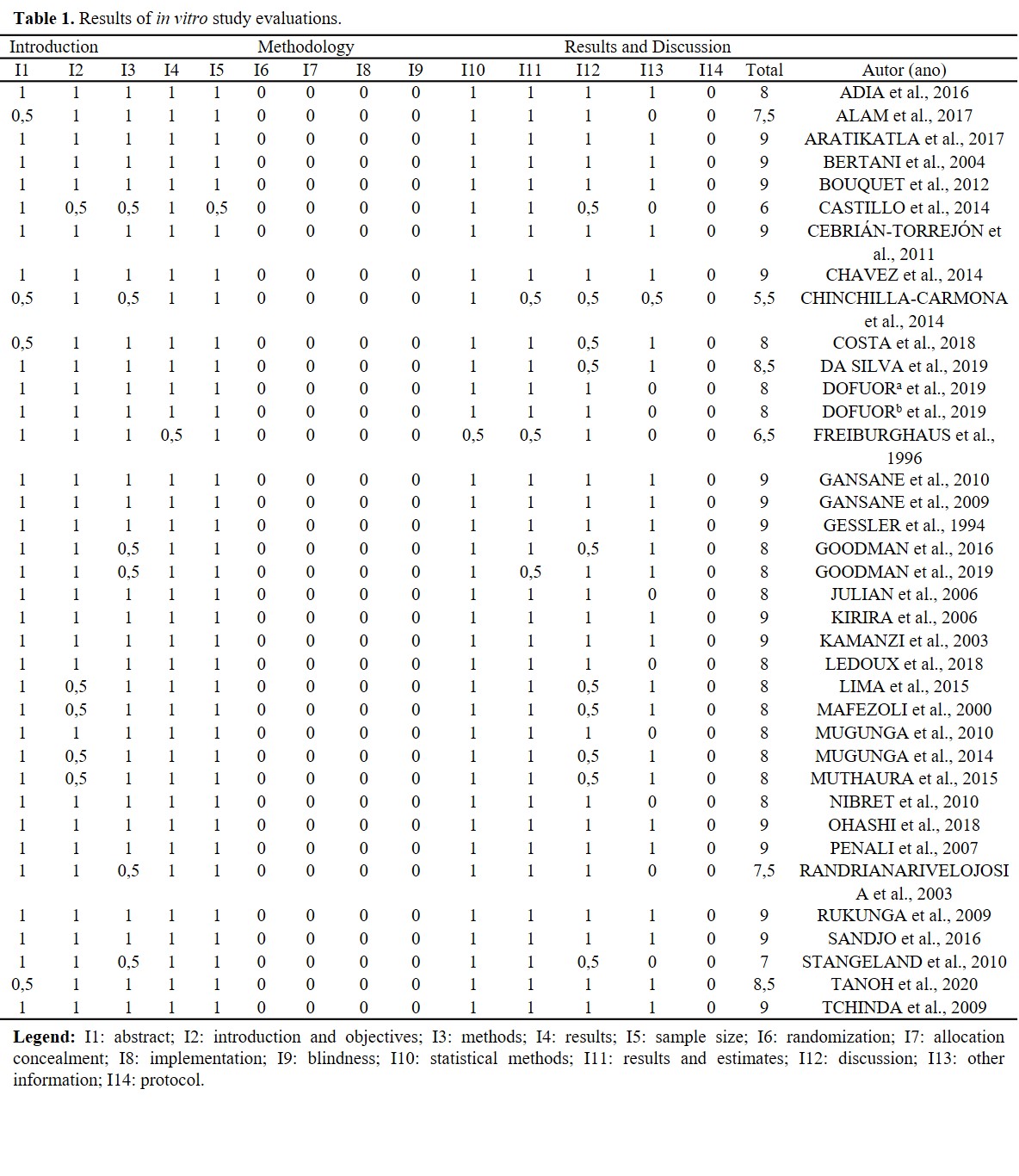

Supplement: Supplementary file 1 [file Image1.JPEG]

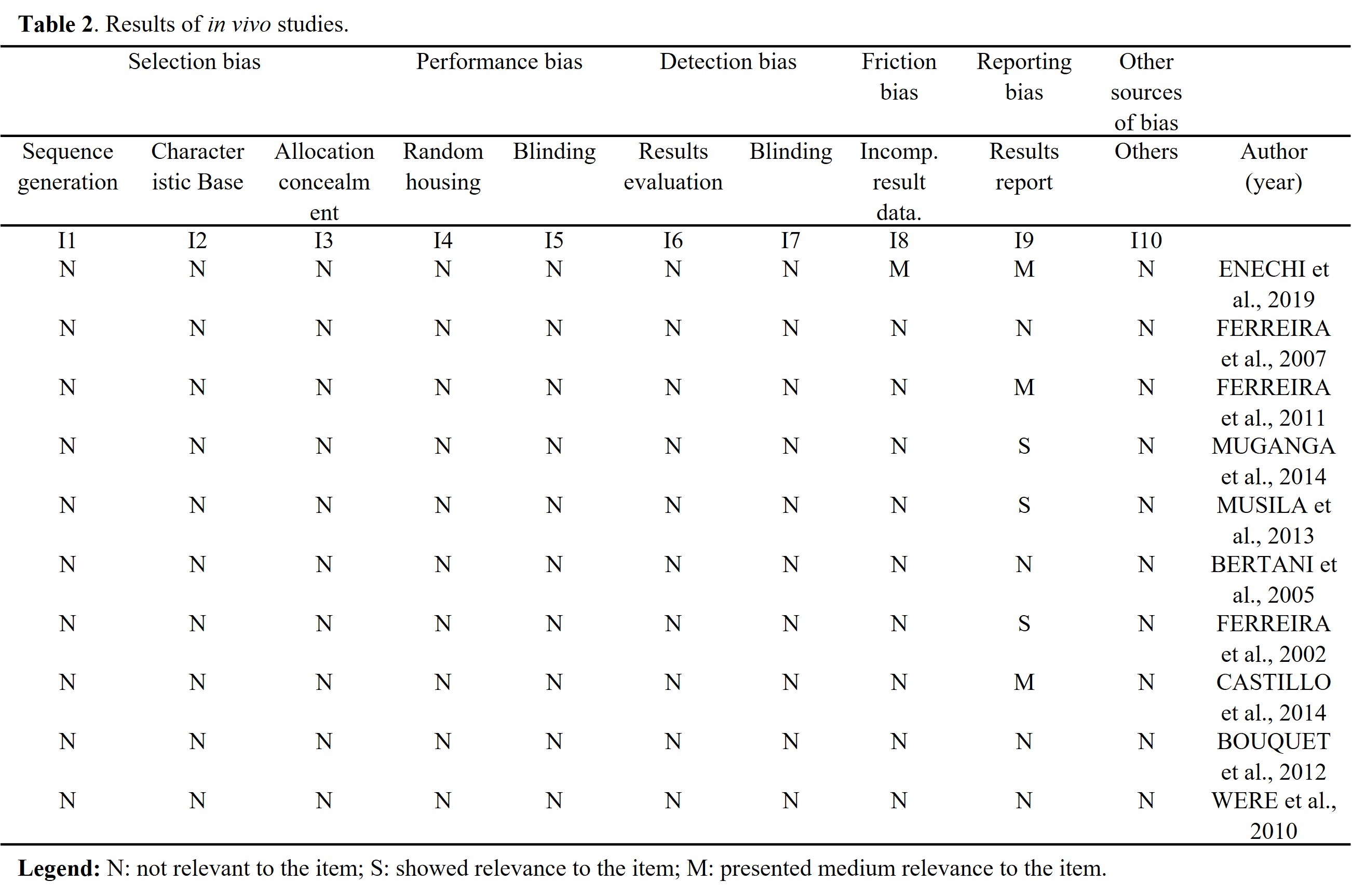

Supplement: Supplementary file 2 [file Image2.JPEG]
